# Evaluation of the Aging of Styrene-Butadiene-Styrene Modified Asphalt Binder with Different Polymer Additives

**DOI:** 10.3390/ma14195715

**Published:** 2021-09-30

**Authors:** Bangwei Wu, Chufan Luo, Zhaohui Pei, Chuangchuang Chen, Ji Xia, Peng Xiao

**Affiliations:** 1 College of Civil Science and Engineering, Yangzhou University, Yangzhou 225127, China; wubw@yzu.edu.cn (B.W.); MZ220190387@yzu.edu.cn (C.L.); MZ220200335@yzu.edu.cn (Z.P.); MZ120200967@yzu.edu.cn (C.C.); MZ120200952@yzu.edu.cn (J.X.); 2 Research Center for Basalt Fiber Composite Construction Materials, Yangzhou University, Yangzhou 225127, China

**Keywords:** SBS, polymer additive, aging property, rheological properties, FTIR

## Abstract

A wide variety of polymer additives have been widely used in recent years. However, the effect of different polymer additives on the durability of asphalt binders has not been investigated thoroughly. To evaluate the aging property of styrene-butadiene-styrene (SBS) asphalt binder with different polymer additives, three polymer modifiers, namely high modulus modifier (HMM), anti-rutting agent (ARA), and high viscosity modifier (HVM), were added to it. First, the Thin Film Over Test (TFOT) and Pressure Aging Vessel (PAV) was performed on the asphalt binders. The rheological properties of the four asphalt binders before and after aging were then checked by the Dynamic Shear Rheometer Test (DSR). The chemical compositions of the asphalt binders were determined by the Fourier Transform Infrared Spectrometer (FTIR) test. Several aging indicators were adopted to reflect the aging degree of the asphalt binders. The results show that when polymer additives are added to the SBS asphalt binder, the complex modulus, storage modulus, loss modulus, and rutting factor substantially increase and the phase angle decreases. All the test parameters become higher after aging. The phase angle of the SBS asphalt binder is the highest at both unaged and aged states, while its other parameters values are the smallest. Moreover, the Carbonyl Aging Indicator (CAI) of SBS with polymer additives becomes lower under both TFOT and PAV conditions, indicating that polymer additives can improve the aging resistance of SBS asphalt, of which HVM modifies the aging resistance best. Complex Modulus Aging Indicator (CMAI) and Storage Modulus Aging Indicator (SMAI) have the best correlation coefficients with CAI, and the two aging indicators can be used to predict the aging degree of polymer modified asphalt binders.

## 1. Introduction

With the increasing traffic load in China, many asphalt pavements may suffer from severe early damage within a few years of completion. People have paid more attention to the durability of asphalt pavements [1,2]. Therefore, in the past decades, in addition to SBS, many other types of modifiers have been used for asphalt mixtures to improve the durability of mixtures, such as the high modulus modifier (HMM) [3], anti-rutting agent (ARA) [3,4], and high viscosity modifier (HVM) [5].

The HMM was initially used in France to improve asphalt mixtures’ rutting resistance and fatigue resistance [3]. The dynamic modulus of high modulus asphalt concrete (HMAC) is about 14 GPa, far higher than that of ordinary asphalt mixtures. Wu [6] investigated the anti-rutting property of HMAC using the wheel track test, and the results show that HMAC has better high-temperature stability than ordinary asphalt mixtures. Lee [7] found that using HMM in asphalt mixtures can increase the stiffness modulus, resulting in a higher deformation resistance of asphalt mixtures.

ARA is also used to modify the anti-rutting property of the asphalt mixture. Chen [8] compared the pavement performance of ARA modified SMA-13 and SBS modified SMA-13 and found that the rutting resistance of the former is far better than that of the latter. A similar conclusion was also drawn by Ulucayli [9]. Sun [10] argued that the elastic component in ARA helped to reduce the shear deformation of asphalt mixtures, resulting in a minor rutting depth in asphalt pavement. Chen [11] thought that the ARA particle could fill the voids in the aggregates, increasing the denseness of asphalt mixtures and bonding adjacent aggregates to resist deformation.

HVM has been widely used for asphalt mixtures in recent years. It can increase the asphalt viscosity significantly. The zero-shear viscosity of HVM modified asphalt at 60 °C can be higher than 40,000 Pa·s [12]. Yang [13] found that HVM modified the adhesion ability of asphalt with mineral aggregate, resulting in asphalt mixtures with better resistance to cracking, deformation, and moisture damage. Tan [14] compared the properties of HVM asphalt and SBS asphalt. The segregation test showed that the compatibility and stability of HVM asphalt were better and the cohesion of the HVM asphalt before and after aging was more stable. The Performance Grade (PG) test results showed that HVM asphalt had a better high and low temperature performance. Li [15] studied the performances of HVM porous asphalt mixtures. After a serial of tests, which included the multi-stress repeated creep test, accelerated fatigue test, temperature sweep, and other tests, Li argued that HVM modified asphalt helped improve the stability and cracking resistance and prevent the loosening of porous asphalt pavements.

It can be seen that types of modifiers improve the pavement performance of asphalt mixtures. However, current research has been conducted into new mixtures, and studies on the aging property of the asphalt binders are lacking. Many studies have proved that the aging property of polymer modified asphalt is more complicated than that of pure asphalt. For polymer-modified asphalt, the aging process includes the aging of the pure asphalt and the degradation and possible chemical reactions of the polymer modifier [16,17,18]. Many researchers have studied the aging behavior of SBS asphalt binder and mixtures, while the durability of other polymer additives has not been studied yet. Cortizo [19] found the degradation of SBS after thermal aging. Sugano [20] insisted that such degradation of SBS decreased the durability of asphalt mixtures. Zhao [21] used Fourier transform infrared spectrum (FTIR) to explore the aging mechanism of SBS asphalt. Zhao found that the chemical structure of SBS changed over the aging process. Thus, the objective of this study is to evaluate and compare the effect of different polymer modifiers on the aging property of the asphalt binder. Three polymer modifiers, namely HMM, ARA, and HVM, were added to the SBS asphalt binder. Short-term aging and long-term aging on the asphalt binders were performed, respectively. A serial of microscopic and macroscopic tests was then conducted to check the aging behavior of the asphalt binders.

## 2. Test Materials

### 2.1. Polymer Additives

**Figure 1:**
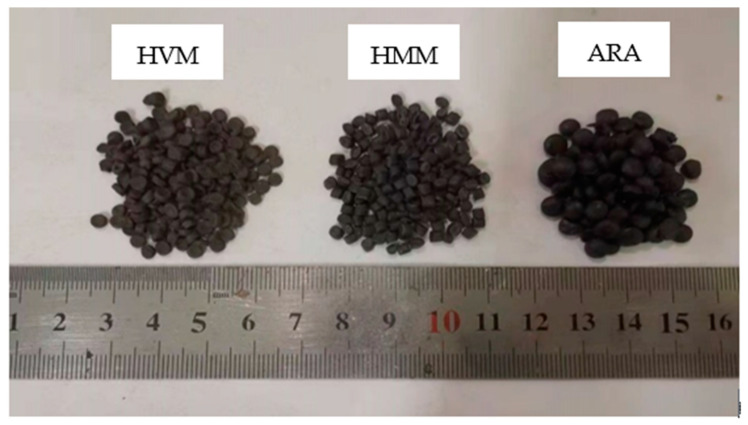
The appearance of the three modifiers.

Three kinds of polymer additives (HVM, HAA, and HMM) were used in this paper. The macro images of the three additives are shown in Figure 1. All three additives were provided by Wanpu Traffic Technology Co., Ltd., Wuxi, China, and they were all black particles at room temperature. The supplier provided the basic properties of the three additives, as presented in Table 1.

### 2.2. Asphalt Binder

In this paper, the SBS asphalt was chosen as the base asphalt binder. It was provided by Tongsha Asphalt Technology Co., Ltd., Nantong, China. The properties of the SBS asphalt were tested according to Chinese specification (JTG E20-2011) Test Standard Methods of Bitumen and Bituminous Mixtures for Highway Engineering [22]. The results are listed in Table 2. The SBS asphalt properties satisfy the requirements in (JTG F40-2004) Technical Specification for Construction of Highway Asphalt Pavements [23]. The polymer additives were then added into the base binder to fabricate a polymer-modified asphalt binder. Thus, a total of four types of asphalt binders were used in this paper, named SBS, HMM-SBS, ARA-SBS, and HVM-SBS for convenience.

According to our team’s previous research results [24], the polymer-modified asphalt binders were prepared by the following steps: Heating the SBS asphalt to 185 °C; adding polymer additive to the SBS asphalt; blending them for 60 min at a speed of 1500 r/min.

## 3. Research Scope and Test Methods

### 3.1. Research Scope

In this paper, the effect of different polymer additives on the aging behavior of SBS asphalt binder was explored. First, short-term aging and long-term aging were performed on the four kinds of asphalt binders. Then, the rheological properties of the four asphalt binders before and after aging were checked, and the chemical compositions of the asphalt binders were also determined by the FTIR. At last, based on other scholars’ research results [25], several indicators calculated by the test results were adopted to reflect the aging degree of the different asphalt binders. These indicators are shown in Table 2.

Many factors affect asphalt aging, and it is the oxidation that mainly causes the aging of the asphalt binder. Therefore, the change in the carbonyl absorption peak can reflect the aging level of the asphalt binder [26]. The Carbonyl Index (CI) calculated by the FTIR test results was used by many researchers to evaluate the aging degree of the asphalt binder [21,27,28]. Thus, this paper used CI as the basic aging indicator, and its correlations with other indicators were analyzed. The calculation method of CI is shown in Table 3.

### 3.2. Test Methods

#### 3.2.1. Aging Methods

In this paper, the short-term aging and long-term aging of the asphalt binder were performed by the Thin Film Over Test (TFOT) and Pressure Aging Vessel (PAV) test, respectively.

The TFOT was used to simulate the thermal oxygen aging of the asphalt binder during storage, transportation, and paving. The PAV test was used to simulate the oxidative aging of asphalt binders during in-service. They were conducted following the steps in ASTM D1754 [29] and ASTM D6521 [30], respectively.

#### 3.2.2. FTIR Test

FTIR adopted in this paper was made by Perkinelmer Instruments Co., Ltd., Shanghai branch, China. This instrument was used in this paper to analyze the changes of chemical functional groups of the asphalt binder before and after aging, thus the mechanism of the difference in the durability of different modifiers could be better understood. The wavenumber used in the test was 600–4000 cm^−1^.

#### 3.2.3. Dynamic Shear Rheometer (DSR) Test

The DSR adopted in this paper is specified in AASHTO T315 [31]. This test method is suitable for determining the phase angle and dynamic shear complex modulus of the asphalt binder. The diameter of the test piece was 25 mm and the thickness was 1 mm. A sinusoidal vibration load with an angular frequency of 10 rad/s was applied to the test piece. The test temperature ranged from 52 °C to 82 °C with an increment of 6 °C/min. The instrument used in this paper was made by TA Instruments Co., Ltd., New Castle, DE, USA.

## 4. Results and Discussion

### 4.1. FTIR Test Results Analysis

#### 4.1.1. FTIR Characteristics before Aging

**Figure 2:**
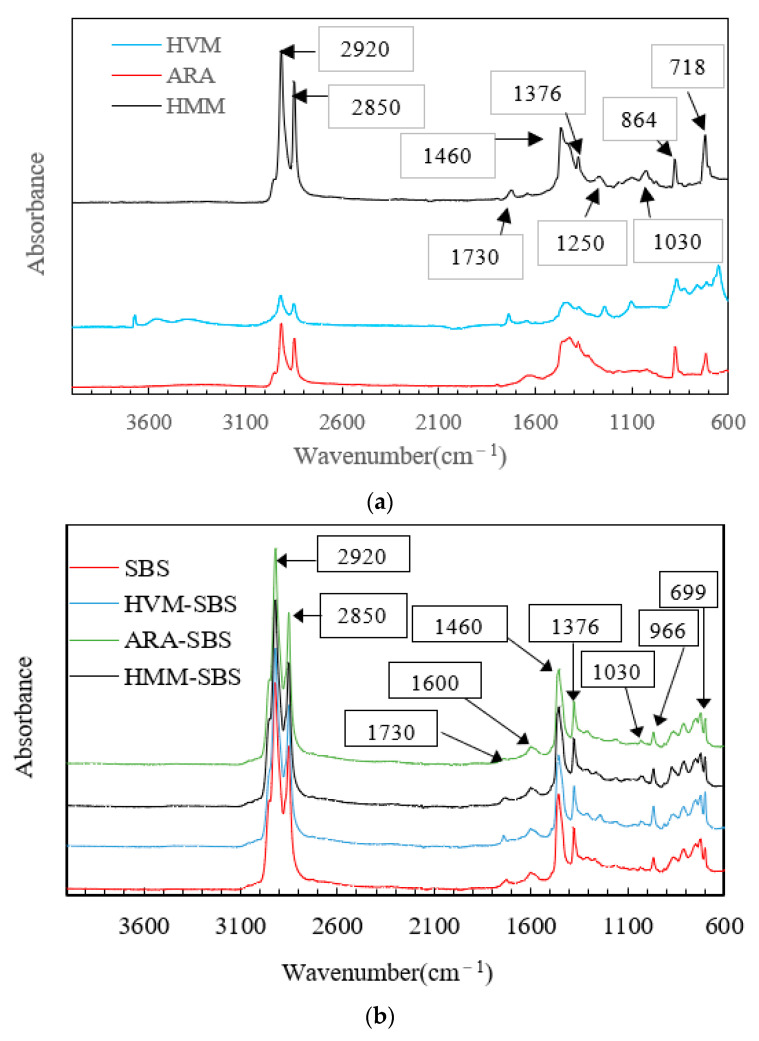
FTIR of polymer additives and asphalt binders before aging: (**a**) polymer additives; (**b**) asphalt binders.

The FTIR pictures of three polymer additives and the four kinds of asphalt binders are presented in Figure 2.

It can be seen from Figure 2a that the FTIR characteristic peaks of the three polymer additives were not the same. For HMM, the absorption peaks mainly appeared at 2920 cm^−1^, 2850 cm^−1^, 1730 cm^−1^, 1460 cm^−1^, 1376 cm^−1^, 1250 cm^−1^, 1030 cm^−1^, 864 cm^−1^, and 718 cm^−1^ etc. The peaks at 2920 cm^−1^ and 2850 cm^−1^ were due to the antisymmetric stretching vibration and symmetric stretching vibration of methylene (CH_2_), separately. The peak at 1460 cm^−1^ was caused by the deformation vibration of the C–H bond. The peak at 1376 cm^−1^ was formed by a specific vibration of methyl (CH_3_). The peak at 864 cm^−1^ was caused by C–C stretch. The peaks at 1030 cm^−1^ and 718 cm^−1^ were due to the S–O stretch and C–S bend, respectively [32,33]. The peaks at 1730 cm^−1^ and 1250 cm^−1^ were the characteristic peaks of esters, which may have been caused by the plasticizer in HMM [34]. HVM showed a similar FTIR characteristic peak to HMM. However, a very slight observable change in peak position was clear in the FTIR spectra of HMM and HVM, indicating that the plasticizer or crosslinker used in HVM and HMM was different. For the FTIR spectra of ARA, there were no characteristic peaks of esters.

Compared with the infrared spectrum of polymer, the infrared spectrum of polymer- asphalt binders (seen as Figure 2b) had several more characteristic peaks, which mainly appeared at 1600 cm^−1^, 966 cm^−1^, and 699 cm^−1^. The 1600 cm^−1^ peak reflected the C=C bond in the benzene ring and the stretching vibration of the C-H bond. The peaks at 966 cm^−1^ and 699 cm^−1^ were the characteristic peaks of the SBS [31]. The FTIR characteristics of the four asphalt binders were similar, indicating that no new chemical functional groups were generated after other polymer additives were added to the SBS asphalt. Other polymer additives did not change the molecular properties of the SBS asphalt binder, and the interaction between the other polymer additives and the SBS was virginly physical.

#### 4.1.2. FTIR Characteristics after Aging

**Figure 3:**
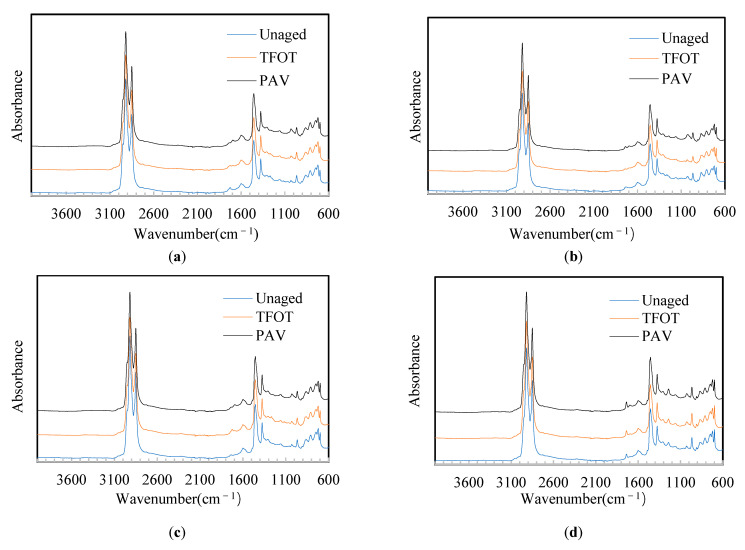
FTIR of asphalt binders after aging: (**a**) SBS; (**b**) HMM-SBS; (**c**) ARA-SBS; (**d**) HVM-SBS.

TFOT and PAV were adopted to age different asphalt binders. The FTIR pictures of asphalt binders after aging are shown in Figure 3.

From Table 4, it can be observed that the aging of the asphalt binders showed the same pattern. The peak areas at 1700 cm^−1^ and 1030 cm^−1^ increased, while the peak areas at 966 cm^−1^ and 699 cm^−1^ decreased, and the peak area at 1376 cm^−1^ was stable. This phenomenon implies that the aging process increases the amount of carbonyl and sulfoxide in the asphalt binder and decreases the amount of butadiene and styrene in the SBS. When the polymer-modified asphalt was aged, some chemical bonds, such as C–C, and C=C, dismantled and reacted with oxygen, or sulfur-based compounds in the asphalt reacted with oxygen to form a new C=O bond and S=O bond [35]. Such chemical reactions resulted in the increase of peak areas at 1700 cm^−1^ and 1030 cm^−1^. Thus, many researchers used the carbonyl index to reflect the aging level of asphalt binders. To further compare the aging level of different asphalt binders, CAI (as shown in Table 3) of the asphalt binders were calculated, and the results are presented in Table 4.

From Table 4, some points can be observed. (1) When the aging pattern changed from TFOT to PAV, the CAI of all the asphalt binders increased. (2) After the SBS asphalt binder was added into other polymer additives, CAI became lower under both TFOT and PAV conditions, indicating that HMM, ARA, and HVM can improve the aging resistance. (3) The ranking of CAI was HVM-SBS < HMM-SBS < ARA-SBS < SBS, indicating that HVM-SBS has the best aging resistance. This phenomenon may be due to the synergistic effect of the esters in the plasticizer and the SBS in the HVM-SBS.

### 4.2. DSR Test Results Analysis

#### 4.2.1. DSR Test Results before Aging

**Figure 4:**
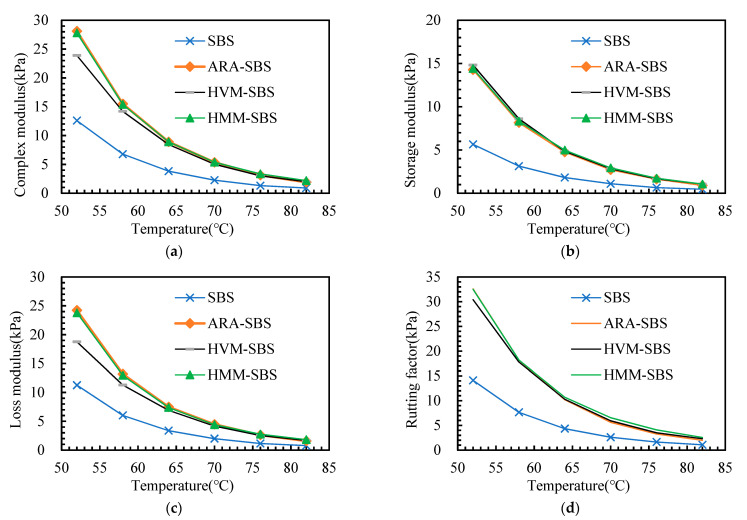

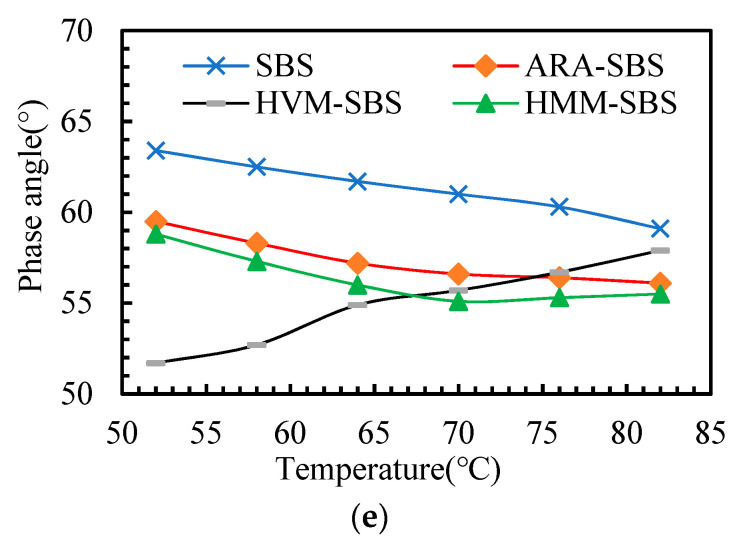
Rheology property of the asphalt binders before aging: (**a**) G*; (**b**) G’; (**c**) G”; (**d**) Rutting factor; (**e**) δ.

According to the DSR test results, the rheology properties of the asphalt binders before aging are given in Figure 4. The complex modulus, storage modulus, loss modulus, phase angle, and rutting factor are compared here.

According to Figure 4, some points can be observed. (1) When polymer additives were added to the SBS asphalt binder, the complex modulus, storage modulus, and loss modulus were substantially increased, and the improvement caused by ARA and HMM was comparable and higher than that of HVM. Moreover, the rutting factor also increased obviously, indicating that polymer additives enhance the rutting resistance of the SBS asphalt binder. (2) The phase angle decreased after the polymer additives were added. A lower phase angle means that there were more elastic components than viscous components in the material. Thus, Figure 4e shows that the addition of polymer additives to the SBS asphalt binder could improve its elasticity, which enhanced the shear-deformation resistance of the SBS asphalt binder. Additionally, the phase angle of the HVM-SBS increased with the temperature, while the other three asphalt binders showed an opposite rule. (3) By comparing Figure 4b,c, it can be observed that the storage modulus of the three polymer asphalt binders was almost the same, while the loss modulus of HVM-SBS was lower than that of HMM-SBS and ARA-SBS. This phenomenon shows that the three polymers had a similar ability to improve the elastic part of SBS asphalt. In contrast, HMM and ARA had a better ability to improve the viscous part of SBS asphalt than HVM. The phase angle refleceds the proportional relationship between the viscous and elastic parts of the material. The different improvements on the elastic and viscous parts of SBS asphalt by the three polymers caused the different phase angles of the three polymer-modified asphalts.

#### 4.2.2. DSR Test Results after Aging

**Figure 5:**
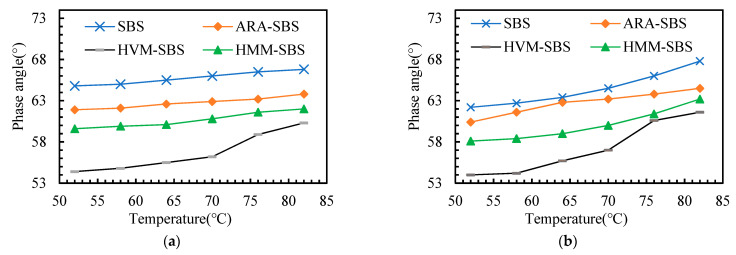
Phase angle of the asphalt binders after aging: (**a**) TFOT; (**b**) PAV.

**Figure 6:**
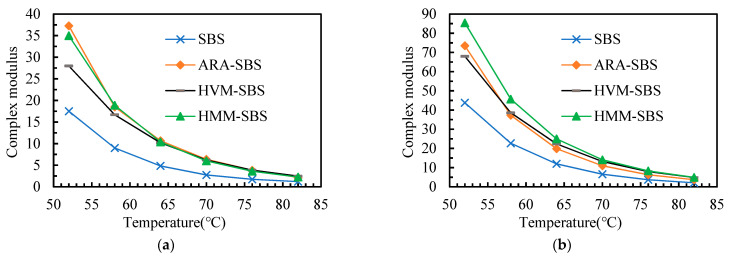
Complex modulus of the asphalt binders after aging: (**a**) TFOT; (**b**) PAV.

The rheology properties of the asphalt binders after aging are analyzed in this section. Considering the fact that (1) the storage modulus, loss modulus, and rutting factor can be calculated with complex modulus and phase angle, (2) the complex modulus, storage modulus, loss modulus, and rutting factor showed a similar change pattern with the aging degree of the asphalt binders deepening, thus this paper takes the complex modulus and phase angle, for instance, to analyze the rheology property of the asphalt binders after aging. The complex modulus and phase angle of the asphalt binders after aging are presented in Figure 5 and Figure 6.

According to Figure 5, several points can be observed. (1) Comparing the phase angle before and after aging, it can be observed that the phase angle of the four kinds of asphalt binders became higher after aging, indicating that the aging process increases the proportion of viscous components in the asphalt binders. Generally speaking, the higher the molecular weight of the polymer, the higher the viscosity. The aging process made the small molecules in the asphalt change to large molecules, causing the proportion of viscous components in the asphalt binder to rise and the phase angle to become higher. (2) The phase angle of the SBS asphalt binder was the greatest at both the short-term aging state and long-term aging state, followed by ARA-SBS, HMM-SBS, and HVM-SBS. This indicates that the polymer additives help to improve the elasticity of the SBS asphalt binder. Aging increased the viscosity of asphalt. Thus, the modification on the elasticity caused by polymer additives decreased the aging sensitivity of the SBS asphalt binder.

From Figure 6, it can be seen that, at both TFPT and PAV conditions, the complex modulus of ARA-SBS and HMM-SBS was higher, the complex modulus of HVM-SBS was lower, and the complex modulus of SBS was the lowest. This phenomenon was similar to the complex modulus before aging. Moreover, comparing the complex modulus before and after aging, it can be found that the complex modulus of asphalt binders increased after both TFOT and PAV. When the aging pattern changed from TFOT to PAV, the increment in complex modulus was more obvious. The aging process made the asphalt binder stiffer, resulting in a greater complex modulus.

The storage modulus, loss modulus, and rutting factor showed the same change pattern as the complex modulus. That is, the deeper the aging degree of the asphalt binder, the higher the values. The aging process increased the storage and loss modulus, resulting in a more excellent rutting resistance of asphalt binders.

#### 4.2.3. Aging Indicators Analysis

**Figure 7:**
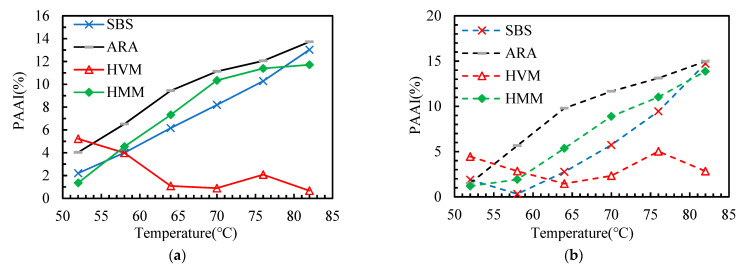
Phase Angle Aging Indicator (PAAI) of the asphalt binders after aging: (**a**) TFOT; (**b**) PAV.

**Figure 8:**
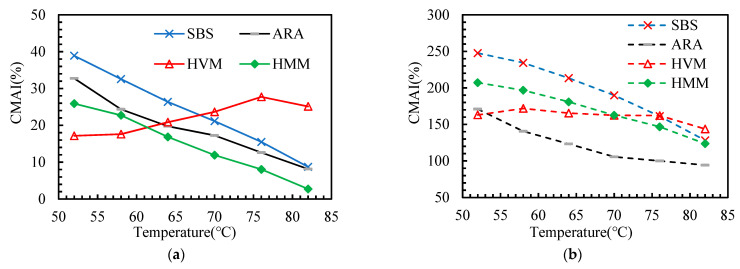
Complex Modulus Aging Indicator (CMAI) of the asphalt binders after aging: (**a**) TFOT; (**b**) PAV.

**Figure 9:**
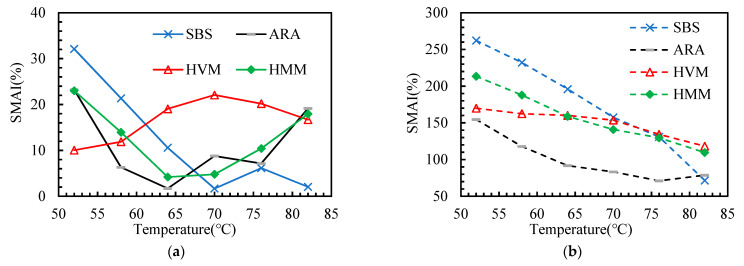
Storage Modulus Aging Indicator (SMAI) of the asphalt binders after aging: (**a**) TFOT; (**b**) PAV.

**Figure 10:**
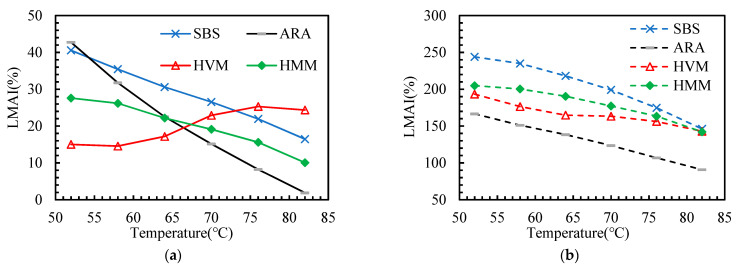
Loss Modulus Aging Indicator (LMAI) of the asphalt binders after aging: (**a**) TFOT; (**b**) PAV.

**Figure 11:**
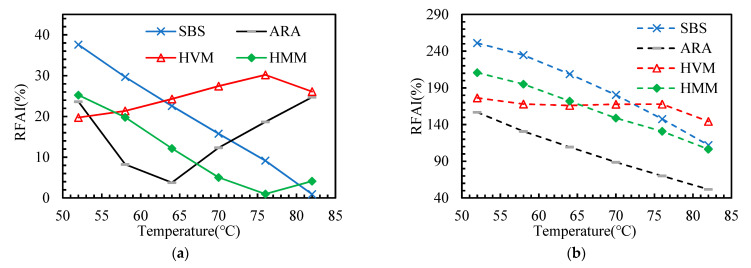
Rutting Factor Aging Indicator (RFAI) of the asphalt binders after aging: (**a**) TFOT; (**b**) PAV.

This paper adopted several aging indicators (as shown in Table 3) to explore the effect of polymer additives on the aging sensitivity of asphalt binders. According to the DSR test results, these indicators are calculated and presented in Figure 7, Figure 8, Figure 9, Figure 10 and Figure 11.

In this paper, the PAAI, CMAI, SMAI, LMAI, and RFAI were calculated to reflect the aging degree of asphalt binders. A higher aging indicator implies a greater aging degree of the asphalt binder. From Figure 7, Figure 8, Figure 9, Figure 10 and Figure 11, some general commonalities can be seen. (1) The result was not the same when the aging degree was evaluated with different aging indicators. For example, the PAAI of ARA-SBS was higher than that of SBS, indicating that ARA-SBS experienced more severe aging. However, the CMAI of ARA-SBS was lower than that of SBS, implying an opposite conclusion. (2) The value of the aging indicators increased significantly when the aging pattern was changed from TFOT to PAV. This phenomenon follows the result of CAI. (3) In general, all the aging indicators, except PAAI of SBS, were higher than that of the other three polymer binders, indicating that polymer additives modify the aging resistance of SBS. However, it is hard to judge which polymer additive modified the aging resistance most. When the improvement on the aging resistance was evaluated with different aging indicators, the evaluation results were not the same. For example, the PAAI of ARA-SBS was higher than that of HMM-SBS, implying that HMM is more beneficial to improve the aging resistance of SBS. However, when this situation was evaluated with SMAI, an opposite conclusion could be drawn. (4) In most cases, the aging indicators of HVM-SBS increased with the temperature, while that of ARA-SBS and HMM-SBS showed the opposite rule.

The above analysis indicates that the aging degree of asphalt binders evaluated by the adopted aging indicators may result in different evaluation results. This phenomenon may be related to a variety of factors. For example, the phase angle and complex modulus of asphalt binders were both temperature dependent. Their temperature sensitivity was strongly related to the molecular weight distribution of various substances within the asphalt [36]. Therefore, when data at different temperatures were used to calculate the aging indicator, it was highly likely that different results would be obtained. Another possible reason is that aging causes changes in the molecular structure of certain substances within the asphalt [37]. These changes have different effects on the rheological indexes, such as phase angle and complex modulus of the asphalt. Thus, different results were obtained when using different aging indicators calculated by different rheological indices to evaluate the aging degree of asphalt binder.

There were six aging indicators in this paper, of which CAI was calculated by FTIR results, and the other five indicators were calculated by DSR results. Because the carbonyl index has proven to be an important indicator of asphalt aging degree [26,27], the aging indicator closely related to CAI can be a helpful indicator to characterize the aging degree of asphalt binders. Therefore, the correlation between CAI and other aging indicators was analyzed. The linear correlation coefficients (denoted by R^2^) calculated by EXCEL software are shown in Table 5.

From Table 5, it can be seen that the correlation coefficients between CAI and other aging indicators declined with temperature in general. The rheological properties of asphalt were temperature dependent. Changes in temperature caused phase changes of certain substances within the asphalt, affecting the rheological properties, resulting in a wide variation in aging indexes at different temperatures. The FTIR of asphalt binders were measured at room temperature, which can lead to a better correlation of rheological property-based aging indexes with CAI at lower temperatures. Moreover, PAAI showed the worst correlation with CAI in all conditions. This may be because PAAI was affected by asphalt aging and was related to other properties of asphalt (such as glass transition temperature). This phenomenon also indicates that PAAI is not applicable to evaluate the aging sensitivity of asphalt, which agrees with the findings of other researchers [25]. When the temperature was lower than 64 °C, CMAI showed the best correlation with CAI, and the highest R^2^ was 0.912. On the contrary, when the temperature was over 64 °C, the correlation coefficient between CAI and LMAI was the greatest. Therefore, the aging degree of polymer-modified asphalt binders can be predicted by CMAI and LMAI.

## 5. Conclusions

In this paper, the aging property of the SBS asphalt binder with different polymer additives were evaluated. The following conclusions can be drawn.

1. The SBS asphalt binder with polymer additives showed a lower CAI under both TFOT and PAV conditions, indicating that polymer additives can improve the aging resistance of the SBS asphalt binder. The ranking of CAI was HVM-SBS < HMM-SBS < ARA-SBS < SBS.
2. When polymer additives were added to the SBS asphalt binder, the complex modulus, storage modulus, loss modulus, and rutting factor were substantially increased, and the improvement caused by ARA and HMM was comparable and higher than that of HVM. The phase angle decreased after the polymer additives were added.
3. The phase angle, complex modulus, storage modulus, loss modulus, and rutting factor of asphalt binders became higher after aging. The phase angle of the SBS asphalt binder was the highest at both unaged and aged states, while its other indicator values were the smallest.
4. CMAI and LMAI had the best correlation coefficients with CAI. The two aging indicators can predict the aging degree of polymer-modified asphalt binders.

In future work, the effect of different polymer additives on the aging behavior of asphalt mixtures should be explored, and the aging mechanism of different polymer-modified asphalt should be studied more thoroughly.

## Figures and Tables

**Table 1:** Properties of additives.

Test Items	HMM	ARA	HVM
Particle size (mm)	2–4	2–3	1–3
Melting point (°C)	120–130	120–150	120–135
Density (g/cm^3^)	0.9–0.98	0.92–0.99	0.9–0.95
Melt index (g/10 min)	5–11	5–11	6–12
Polymer content (%)	≥95		
Exterior	Black solid particles		

**Table 2:** Properties of SBS asphalt.

Index	Results	Requirements
Penetration at 25 °C (0.1 mm)	68	60~80
Penetration Index	0.4	≮−0.4
Ductility at 5 °C (cm)	45	≮30
Softening point (°C)	62	≮55
Viscosity at 135 °C (Pa·s)	1.4	≯3.0
Elastic recovery at 25 °C (%)	76	≮65

**Table 3:** Aging indicators in this paper.

Indicators	Calculation Methods
Phase Angle Aging Indicator (PAAI) (%)	100×|(δ_aged_ − δ_unaged_)|/ δ_unaged_
Complex Modulus Aging Indicator (CMAI) (%)	100×|(G* _aged_ − G* _unaged_)|/ G* _unaged_
Storage Modulus Aging Indicator (SMAI) (%)	100×|(G’_aged_ − G’ _unaged_)|/ G’ _unaged_
Loss Modulus Aging Indicator (LMAI) (%)	100×|(G”_aged_ − G” _unaged_)|/ G” _unaged_
Rutting Factor Aging Indicator (RFAI) (%)	100 × |(G*_aged_/sinδ − G* _unaged_/sinδ)|/ (G*_aged_/sinδ)
Carbonyl Aging Indicator (CAI) (%)	CAI = 100 × |(CI _aged_ − CI _unaged_)|/ CI _unaged_;

G* is the complex shear modulus; δ is the phase angle; G’ is the storage modulus, G’ = G*cosδ; G” is the loss modulus, G” = G*cosδ; CI = A1700 cm^−1^/A1376 cm^−1^; A Xcm^−1^ is area at X cm^−1^ peak of the FTIR figure.

**Table 4:** CAI of the asphalt binders.

Binder Type	SBS	HMM-SBS	ARA-SBS	HVM-SBS
TFOT	467.8	385.4	438.5	330.1
PAV	3798.6	2441.2	2772.6	1450.5

**Table 5:** The correlation coefficients between CAI and other aging indicators.

Indicators	R^2^ at the Following Temperatures (°C)					
	52	58	64	70	76	82
PAAI	0.237	0.374	0.006	0.006	0.067	0.189
CMAI	0.912	0.851	0.815	0.759	0.692	0.661
SMAI	0.886	0.828	0.747	0.677	0.552	0.459
LMAI	0.843	0.847	0.843	0.801	0.748	0.699
RFAI	0.871	0.836	0.779	0.686	0.645	0.469

## Data Availability

The data presented in this study are available on request from the corresponding author.
